# OM14 is a mitochondrial receptor for cytosolic ribosomes that supports co-translational import into mitochondria

**DOI:** 10.1038/ncomms6711

**Published:** 2014-12-09

**Authors:** Chen Lesnik, Yifat Cohen, Avigail Atir-Lande, Maya Schuldiner, Yoav Arava

**Affiliations:** 1 Department of Biology, Technion—Israel Institute of Technology, Haifa 3200003, Israel; 2 Department of Molecular Genetics, Weizmann Institute of Science, Rehovot 7610001, Israel

## Abstract

It is well established that import of proteins into mitochondria can occur after their complete synthesis by cytosolic ribosomes. Recently, an additional model was revived, proposing that some proteins are imported co-translationally. This model entails association of ribosomes with the mitochondrial outer membrane, shown to be mediated through the ribosome-associated chaperone nascent chain-associated complex (NAC). However, the mitochondrial receptor of this complex is unknown. Here, we identify the *Saccharomyces cerevisiae* outer membrane protein OM14 as a receptor for NAC. *OM14Δ* mitochondria have significantly lower amounts of associated NAC and ribosomes, and ribosomes from NAC[*Δ*] cells have reduced levels of associated OM14. Importantly, mitochondrial import assays reveal a significant decrease in import efficiency into *OM14Δ* mitochondria, and OM14-dependent import necessitates NAC. Our results identify OM14 as the first mitochondrial receptor for ribosome-associated NAC and reveal its importance for import. These results provide a strong support for an additional, co-translational mode of import into mitochondria.

## Abstract (web summary)

Mitochondrial proteins can be imported post-translationally; however, a role for co-translational import has recently provoked renewed interest. Lesnik *et al.* identify OM14 as a mitochondrial ribosome receptor required for efficient co-translational import of mitochondrial proteins.

## Graphical abstract

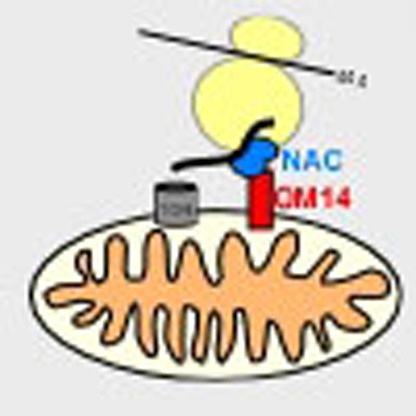

Mitochondria contain several hundred proteins that are critical for their diverse functions. Most of these proteins are encoded in the nucleus, synthesized in the cytoplasm and imported into mitochondria through their import complexes (TOM and TIM complex)^1^. The import process of most mitochondrial proteins can occur following their complete synthesis in the cytosol (that is, post-translationally) and the mechanisms for such a process are well established (reviewed in refs 2, 3, 4). An additional model, in which proteins are imported while being translated (co-translationally) was proposed ~40 years ago following the detection of translationally active ribosomes near the mitochondrial outer membrane^5^,^6^. This model was abandoned for many years and was recently revived following diverse observations (reviewed in refs 7, 8). In particular, genome-wide microarray analyses revealed that many messenger RNAs are associated with the mitochondrial outer membrane, and advanced microscopic techniques provided important confirmation of these results^9^,^10^,^11^,^12^. These mRNAs were proposed to be translated locally (that is, near mitochondria), thereby positioning the emerging polypeptide chain in close proximity to the TOM complex and facilitating import. Indeed, treatments with various translation inhibitors or changes in various coding domains affected mRNA association, thereby supporting the hypothesis that mRNA association is linked to translation^10^,^12^,^13^,^14^,^15^. Furthermore, we have shown that deletion of Tom20, a protein receptor for incoming precursor proteins, affects mRNA association^10^. This result provides an important link between protein import and mRNA association, which can be explained by a co-translational mode of import. Yet, direct support for co-translational import is still scarce. Moreover, the proteins that may be involved in such a process are largely unknown.

The nascent chain-associated complex (NAC) is a ribosome-associated chaperone that is conserved from yeast to human^16^. It binds ribosomes in close proximity to the protein exit tunnel, and interacts with newly synthesized proteins as they exit the ribosome^17^. NAC was shown to support protein transport to various cellular destinations, including mitochondria^18^,^19^. The α-subunit of NAC (the Egd2 protein in the budding yeast *S. cerevisiae*) can dimerize either with a β1 (Egd1 in yeast) or β3 (Btt1 in yeast) subunit, thereby forming two different heterodimers^20^,^21^,^22^. One of the two NAC heterodimers was shown to associate preferentially with nascent chains of mitochondria-targeted ribosome–nascent-chain complexes (RNCs)^22^. *In vitro* studies have shown that NAC can promote protein import when preformed RNCs are mixed with purified mitochondria^23^,^24^ and deletion of NAC subunits in yeast cells reduced ribosomal association with mitochondria^25^. Furthermore, *in vivo* studies have shown that co-translational import of mitochondrial fumerase is lower upon NAC deletion^26^. Thus, NAC is a mediator of ribosomes’ association with mitochondria, and a critical player in co-translational import. Notably, association of NAC with mitochondria was shown to necessitate a mitochondrial receptor^24^, however, such a receptor has not yet been identified. Furthermore, the two trivial candidates (the protein receptors Tom20 and Tom70) were specifically excluded^24^.

In this work, we perform a genome-wide protein complementation screen for proteins that interact with either NAC subunit. We find the mitochondria outer membrane protein OM14 to be a positive partner. OM14 appeared to interact with NAC in all eight different types of screen that we performed. Co- immunoprecipitation analyses confirmed these results. Furthermore, the mitochondrial fraction from OM14-deleted cells had significantly reduced levels of associated NAC and ribosomes. Complementary to this result, ribosomes from NAC-deleted cells had reduced OM14 association. Through import assays into mitochondria, we show that OM14-deleted mitochondria have reduced co-translational import efficiency, and this role in import is exerted through NAC. Thus, OM14 is a receptor for ribosome-associated NAC, thereby coordinating localized translation and import into the mitochondria.

## Results

### NAC interacts with OM14

**Figure 1:**
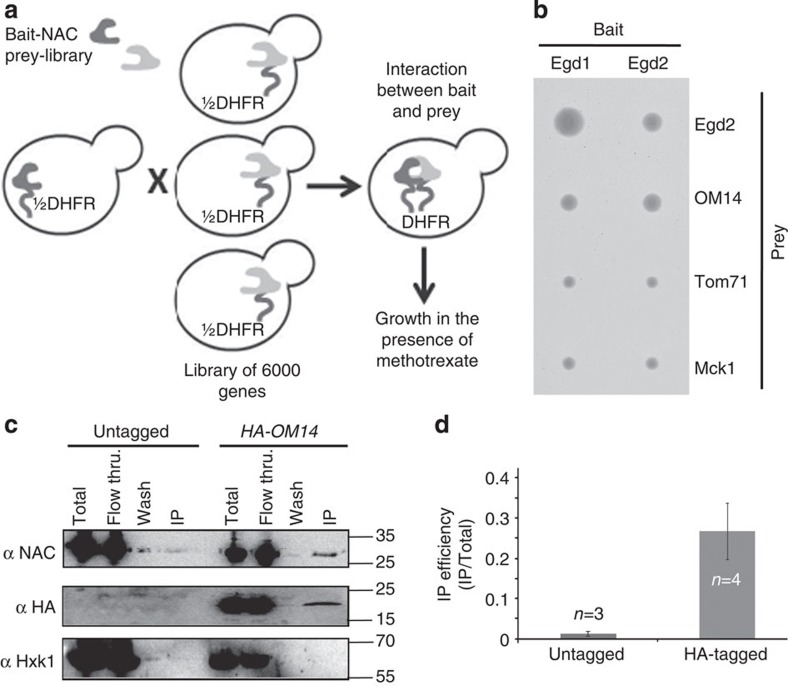
OM14 interacts with NAC. (**a**) Scheme of the PCA screen. Yeast strain expressing a bait protein fused to the amino (N)-terminal half of DHFR is mated with a library of yeast strains, each expressing a prey ORF fused to the carboxy (C)-terminal half of DHFR. Mating is done automatically, and the resulting cells are plated in an ordered manner of methotrexate-containing plates. Proximity of the two DHFR halves allows cells’ growth in the presence of methotrexate. Colonies size are determined with Balony program after 3 days of growth and compared with cells that express only the DHFR halves. (**b**) Example of selected interactions with NAC members, Egd1 and Egd2. Egd2 is known to heterodimerize with Egd1, Tom71 serves as a control for other mitochondria outer membrane proteins and Mck1 is a cytosolic kinase that serves as a standard negative control in such assays. (**c**,**d**) Co-immunoprecipitation analysis. Strains expressing either untagged or HA-tagged OM14 were subjected to immunoprecipitation (IP) with anti-HA beads, and samples from different steps of isolation were analysed by western blot with the indicated antibodies. Total indicates sample before mixing with the beads, Flow Thru. (flow through) is a sample from the unbound material, Wash indicates sample from the last wash of beads and IP is the eluted material from the beads. The histogram (**d**) presents the average results of NAC co-IP efficiency, calculated as the IP/input ratio for NAC divided by HA-OM14 ratio. Data are from at least three independent biological replicates (*n*), each entailing the entire procedure described above, from cell growth to western analysis. Error bars represent s.e.m. *P* value=0.028 (independent-samples one-sided *t*-test). Normal distribution was verified by standard tests (either Shapiro–Wilk or Kolmogrov–Smirnov).

The NAC complex was previously shown to support association of ribosomes with mitochondria in a manner that necessitated a mitochondrial receptor^24^,^25^,^27^. To identify a possible receptor for NAC (and hence ribosomes) on the mitochondrial outer membrane, we performed a genome-wide protein complementation assay (PCA) by utilizing the split dihydrofolate reductase (DHFR) system^28^ (Fig. 1a). In this system, a haploid yeast strain expressing NAC subunit fused to one half of DHFR protein (bait) is mated into a library of ~6,000 strains, each expressing a different yeast protein fused to the other half of DHFR (prey). Interaction between the bait and a candidate prey brings the two DHFR halves to a close proximity and renders the cells resistant to methotrexate. We performed such a screen with either the α-subunit of NAC (Egd2) or the β-subunit (Egd1) as baits. Each subunit was expressed in either a or α mating type with either N′ or C′ halves of DHFR, respectively) and mated with a library of the reciprocal mating type. Colonies were then grown on either glucose or galactose as carbon source. This resulted in eight different screens (two baits × two mating types × two growth media). Diploids of each screen were allowed to grow for 3 days on methotrexate and colony size was measured with the Balony program^29^. The list of strains that passed our threshold of confidence (were larger than 150 pixels) is shown in Supplementary Table 1. The only mitochondrial membrane protein that appeared in the largest 100 colonies was OM14, an outer membrane protein with an unknown function^30^. The high ranking of OM14 occurred in each of the eight screens, corroborating the validity of the interaction (Supplementary Table 1). These results were re-confirmed by mating only these strains and their controls (Fig. 1b).

To validate the PCA results by an alternative approach, we performed co-immunoprecipitation analysis (co-IP). OM14 gene was tagged with HA through homologous recombination. Cells expressing an HA-tagged OM14 were lysed in the presence of a detergent (to disassociate OM14 from mitochondrial membranes), and immunopurified with anti-HA beads. Western analysis with antibodies recognizing the Egd2 subunit of NAC revealed significant amounts at the IP fraction (Fig. 1c,d). This co-purification is not due to non-specific binding to the beads because it does not present when a strain expressing untagged OM14 was used or when an unrelated protein (HXK1) was tested.

In summary, the systematic PCA analysis uncovered a novel physical interactor for the NAC on the surface of mitochondria. Such an interaction could mediate NAC targeting to the surface of mitochondria.

### Importance for association of ribosomes with mitochondria

**Figure 2:**
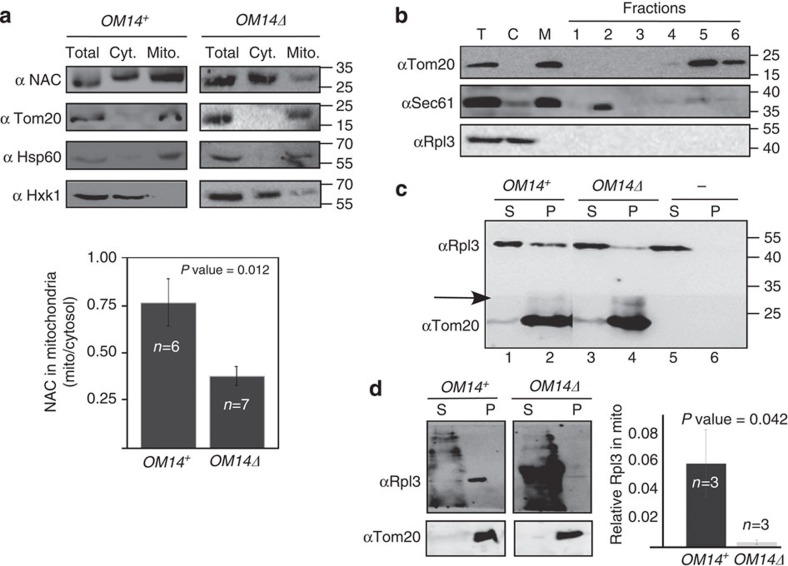
Decreased NAC and ribosomes association with *OM14Δ* mitochondria. (**a**) Cells were subjected to cellular fractionation and samples from the cytosolic (Cyt.) or mitochondrial (Mito.) fractions were subjected to western analysis with the indicated antibodies. The histogram presents the average of the ratio between NAC signals in the two fractions from several independent biological repeats (*n*), each entailing the entire procedure, from cells’ growth to western analysis. Error bars represent s.e.m. *P* value was calculated by independent-samples one-sided *t*-test. Normal distribution was verified by standard tests (either Shapiro–Wilk or Kolmogrov–Smirnov). (**b**) Purification of mitochondria depleted of ER or ribosomes. Following translational arrest, cell lysate (T) was prepared in the presence of EDTA and separated into cytosolic (C) and membrane (M) fractions by differential centrifugation. The M fraction was further separated through a sucrose gradient into six fractions. Aliquot from each fraction was subjected to western analysis with the indicated antibodies. (**c**) ER and ribosome-depleted mitochondria were isolated either from OM14^+^ or *OM14Δ* cells, incubated with ribosomes and subjected to centrifugation. Sup (S) and pellet (P) samples were analysed by western blot with the indicated antibodies. The arrow indicates the horizontal line in which the membrane was cut, to allow simultaneous incubation with the indicated primary antibodies. Also, unrelated lane was cropped between lanes 2 and 3. (**d**) Ribosomes with a nascent chain of mitochondria protein (MDH1) were incubated with mitochondria and separated to S and P, as above. Samples were analysed by western blot with the indicated antibodies. The presented panels are from the same image, that was cropped to remove unrelated lanes. Histograms present the results of three independent biological repeats and statistics were calculated as above. *Y* axis is the (P/P+S) signal for Rpl3, normalized to the signal of Tom20.

We next wished to determine if the interaction between NAC and OM14 is important for the association of ribosomes with mitochondria. To this end, we isolated a crude mitochondrial fraction from cells either expressing or devoid of OM14, and examined the amounts of NAC associated with this fraction by western analysis. Indeed, there was a significant reduction in the amount of NAC that appeared in the mitochondrial fraction of cells deleted for OM14. This is not due to reduced amounts of mitochondria in the *OM14Δ* sample, as the levels of another outer membrane protein (Tom20) or a luminal protein (Hsp60) appear similar (Fig. 2a). We note that there are still detectable levels of NAC in *OM14Δ* mitochondria fraction. These may be due to some impurities (for example, ER components) in this fraction, or due to activity of other receptors that compensate for OM14 deletion. A marker for ribosome association (Rpl3) appeared almost unchanged (not shown), probably because our crude mitochondrial fraction contains also significant amount of ER-bound ribosomes, which are not affected from OM14 deletion.

To test for impact on ribosomal association with mitochondria more precisely, we established an association protocol that utilize highly purified mitochondria (Fig. 2b) and ribosomes (see Methods). Mitochondria were separated from ER by density centrifugations through a sucrose gradient (Fig. 2b). To ensure that the only NAC source is the ribosome-associated one, mitochondria samples were purified from cells deleted of NAC. We note that this purification protocol necessitates disassembly of ribosomes (routinely imposed by the Zymolyase treatment or by the addition of ethylenediaminetetraacetic acid (EDTA))^31^, leading to the absence of ribosomes in the mitochondria fraction. Any attempt to maintain ribosomes with mitochondria hinders the separation of the ER from mitochondria^31^. Ribosome-stripped mitochondria from either OM14^+^ or *OM14Δ* cells were incubated with ribosomes that were cleared from heavy complexes (for example, mitochondria or microsomes) through differential centrifugation. Mixed mitochondria and ribosomes were then centrifuged at 10,000 *g* (Fig. 2c), as under these conditions ribosomes are maintained in the supernatant, unless mitochondria are present (Fig. 2c, lanes 5 and 6). Quantitation of ribosomes’ sedimentation (by the Rpl3 signal normalized to the Tom20 signal), reveals at least twofold higher ribosomal association with mitochondria that contain OM14 (Fig. 2c).

We reasoned that the twofold higher association might be even higher if ribosomes were loaded with a mitochondrial targeting sequence (MTS). We, therefore, purified ribosomes from an *in vitro* translation reaction, that was performed in the presence of a transcript encoding a mitochondria protein (MDH1t). This transcript did not contain a stop codon, to improve ribosomes stalling on the transcript^24^,^32^ (see also Methods). Indeed, ribosomes association with *OM14Δ* mitochondria was negligible, and significantly higher in OM14^+^ mitochondria (Fig. 2d; *P* value=0.042, independent-samples one-sided *t*-test). Thus, an outer membrane-assembled OM14 is capable of increasing ribosome association with isolated mitochondria.

**Figure 3:**
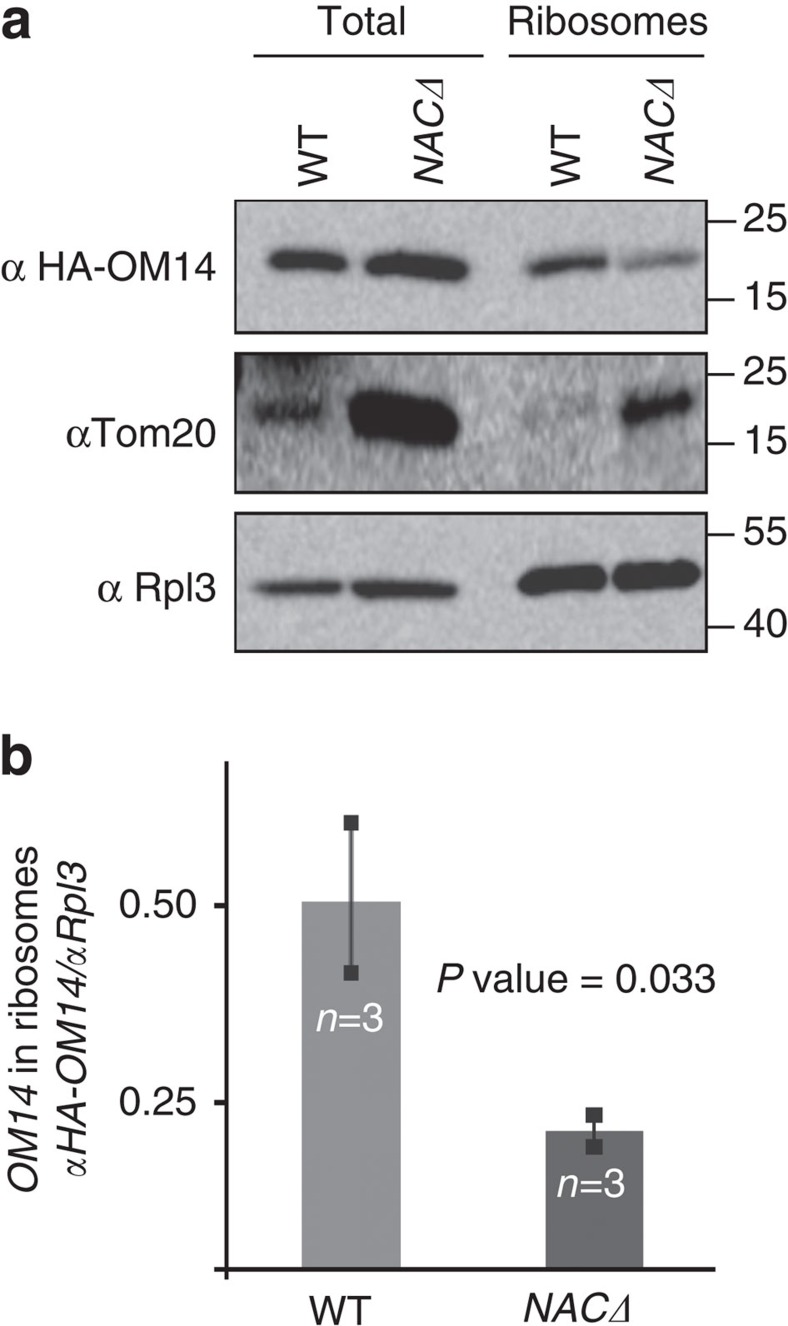
Lower OM14 association with *NACΔ* ribosomes. Ribosomes were isolated from cells either expressing or deleted of NAC genes. A sample before isolation (total) or after centrifugation steps (ribosomes) was analysed by western analysis with the indicated antibodies. The histogram presents the average ratio of signals from several independent biological repeats (*n*) each entailing the entire procedure, from cells’ growth to western analysis. Error bars represent s.e.m. *P* value was calculated by independent-samples one-sided *t*-test. Normal distribution was verified by standard tests (either Shapiro–Wilk or Kolmogrov–Smirnov).

To substantiate that OM14 association is indeed with ribosomes, we performed the complementary analysis, in which we isolated ribosomes and tested for OM14 association. Yeast cells were lysed in the presence of detergent to disassemble mitochondria membranes and ribosomes were isolated by differential centrifugation. The association of HA-tagged OM14 was tested by western analysis (Fig. 3). We found that significant amounts of OM14 co-sedimented in the ribosomal pellet prepared from WT cells, unlike other mitochondrial membrane proteins such as Tom20. Importantly, when ribosomes were devoid of NAC (that is, isolated from *NACΔ* strain (*egd1Δegd2Δ*)), significantly lower amounts of OM14 co-sedimented. This is not due to lower amounts of ribosomes in this preparation, as the levels of ribosomal marker (Rpl3) appear similar. Intriguingly, the levels of Tom20 appear to be highly induced in *NACΔ* cells, while the levels of Rpl3 appear similar. This may suggest a compensation mechanism that is activated upon the absence of this ribosome-associated complex. Overall, these results confirm that NAC interacts specifically with OM14. More important, they reveal that NAC–OM14 interaction occurs while NAC is associated with ribosomes, consistent with NAC being almost exclusively ribosome-associated^19^,^33^.

### OM14 supports import to mitochondria

**Figure 4:**
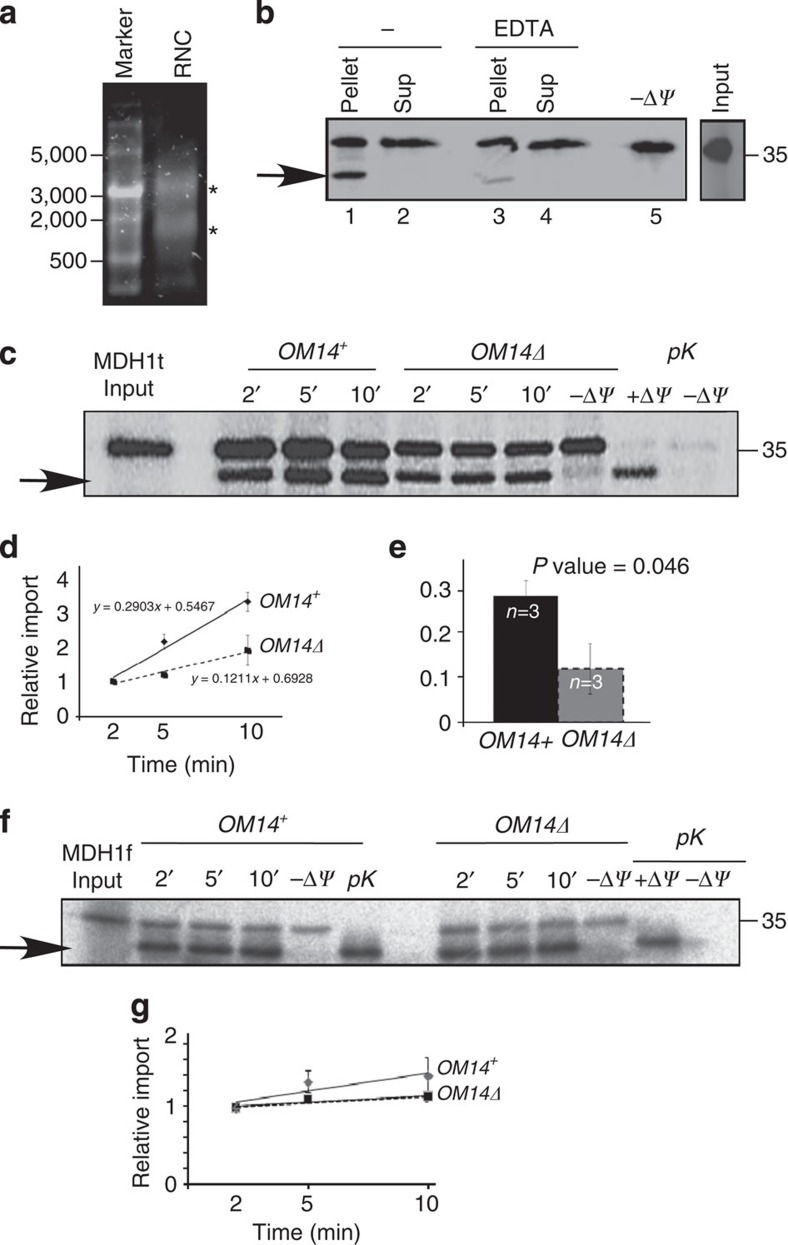
*OM14Δ* mitochondria exhibit lower co-translational import efficiency. (**a**–**e**) Co-translational import of MDH1t precursor: (**a**) Stalled ribosome–nascent chains complexes were isolated by centrifugation through a sucrose cushion and mixed with highly purified mitochondria. Ethidium bromide staining of pelleted RNCs is presented. Bands at the sizes of rRNAs of the small and large subunit are indicated by asterisks. (**b**) RNCs labelled by ^35^S-Met were mixed with highly purified mitochondria (fraction five in Fig. 2b) and incubated for 5 min with or without 20 mM EDTA. Mitochondria were then isolated by centrifugation and proteins associated with mitochondria (pellet) or not (sup) were resolved on PAGE. The arrow indicates a protein that was inserted into the mitochondria and cleaved (hence it is shorter). This band is not detected in the (−Δ*Ψ*) control reaction in which the membrane potential was diminished. Input panel is a sample from the protein labelling reaction before mixing with mitochondria. (**c**) MDH1t RNCs were mixed with highly purified mitochondria from *OM14*^*+*^ or *OM14Δ* cells. At the indicated time points, an aliquot was set aside and resolved on PAGE and phosphorimager. Control reactions included the addition of 1 μg ml^−1^ of valinomycin (−Δ*Ψ*) during a 10 min reaction or addition of proteinase K (pK) at the end of a 10 min import reaction, to remove non-imported bands. (**d**) Import assays were repeated three times (*n*), with three time-point measurements in each. Every repeat entailed a new mitochondria prep and a new RNC prep. The average value and s.e.m. for each time point is presented. Graphs are the best-fit linear slope. (**e**) The histogram presents the average and s.e.m of the best-fit slopes from the three (*n*) independent experiments. *P* value was calculated by independent-samples one-sided *t*-test. Normal distribution was verified by standard tests (either Shapiro–Wilk or Kolmogrov–Smirnov). (**f**,**g**) Post-translational import: Full-length MDH1 (MDH1f) was synthesized from its normal ORF in a rabbit reticulocyte lysates with ^35^S-Met. Import assays were performed as above, with the indicated controls. Panel **e** presented the results of three biological repeats, with three time-point measurements in each. Every repeat entailed new mitochondria prep and a new protein synthesis reaction. The average value and s.e.m. for each time point is presented. Graphs are the best-fit linear slope. No statistically significant difference is apparent between the linear best fits of graphs (*P* value=0.33, independent-samples one-sided *t*-test).

The functional role of OM14 is unknown. Considering its interaction with NAC, we sought to determine if it has a role in co-translational protein import to the mitochondria. We generated ribosome–nascent chain (RNC) complexes in a rabbit reticulocyte lysate by utilization of a truncated MDH1 construct (MDH1t) that induce ribosome stalling at the end of the mRNA^24^,^32^. RNCs were isolated from the lysate by centrifugation through a sucrose cushion^34^,^35^, and indeed rRNA of both ribosomal subunits was detected in the pellet (Fig. 4a). To confirm the functionality of these RNCs, they were mixed with highly purified mitochondria (that is, depleted of ribosomes or ER marker)^36^ (Fig. 2b), and import of MDH1t was allowed (Fig. 4b); imported MDH1t appears as a shorter protein (due to cleavage of the leader sequence) when proteins are resolved on gel. This shorter band does not appear when membrane potential is diminished by the addition of valinomycin (−Δ*Ψ*) and is protected from cleavage by proteinase K (see also Fig. 4c). About 60% of the protein appeared to co-sediment with mitochondria, half of it appeared as a shorter band in the gel. This reveals that the RNCs are functional and permit protein association and insertion into mitochondria, at least up to a point that allows cleavage by the mitochondrial peptidase. Importantly, when the same experiment was performed in the presence of EDTA, which disassembles ribosomes, a significant reduction in import was observed (Fig. 4b). This strongly suggests that import occurs while ribosomes are in complex with the nascent chain.

To test if OM14 contributes to co-translational import, we performed time-course import assay with RNCs and mitochondria purified from *OM14Δ* strain or its parental strain. These strains were also deleted of NAC, to ensure that the only source of NAC is from the RNCs. More than twofold decrease in import efficiency was observed in mitochondria deleted of OM14 (Fig. 4c–e) compared with mitochondria that contain OM14. The slope of the best-fit linear curve, which is a proxy to import rate, is more than twice lower in *OM14Δ* mitochondria compared with OM14^+^ cells (Fig. 4e). We note that OM14 is not essential for import, as even in its absence there is some import of MDH1t. This is not surprising as OM14 is not a core import component, thus is more likely to have an auxiliary role. This is consistent with OM14 being non-essential for yeast viability, even under respiration-dependent conditions (Supplementary Fig. 1)^30^.

We further tested whether OM14 affects import of fully synthesized proteins (that is, post-translationally). For that, we synthesized MDH1 from its full open reading frame (ORF; MDH1f), leading to a complete synthesis of the protein, without ribosome stalling^37^. As these import reactions do not rely on functional RNCs, their efficacy and quality is much higher (Fig. 4f). The mitochondrial preps that are used for the post-translational import assay are the same as the ones that are used for the co-translational assays, thus these results validate the integrity of the mitochondria and their import potency. Comparing the post-translational import of OM14^+^ to *OM14Δ* mitochondria from multiple experiments, revealed an insignificant small decrease in import (*P* value=0.33, independent-samples one-sided *t*-test). Thus, OM14 impact appears to be specific for the co-translated MDH1. Intriguingly, when import reactions were performed with a commonly used chimeric Su9-DHFR protein (Supplementary Fig. 2), a more significant decrease was observed in *OM14Δ* mitochondria. Although this may suggest that OM14 have a differential impact on post-translational import of proteins, a larger repertoire of proteins, preferably native ones need to be tested. Overall, however, these data reveal that OM14 contributes to co-translational import to yeast mitochondria.

### OM14 role in import is exerted through NAC

To validate that OM14 necessitates NAC to exert its function, we assembled import complexes that lack NAC, and then introduced NAC to the import assay. NAC-depleted RNCs were obtained by centrifugation of the RNCs through a sucrose cushion supplemented with high salt concentration (0.8 M KAc; ref. 34). Western analysis with anti-αNAC confirmed that NAC is depleted from the RNC pellet and maintained in the supernatant (Supplementary Fig. 3a). Consistent with previous studies, these NAC-depleted RNCs showed much lower import efficiency into mitochondria (Supplementary Fig. 3b–d)^24^.

**Figure 5:**
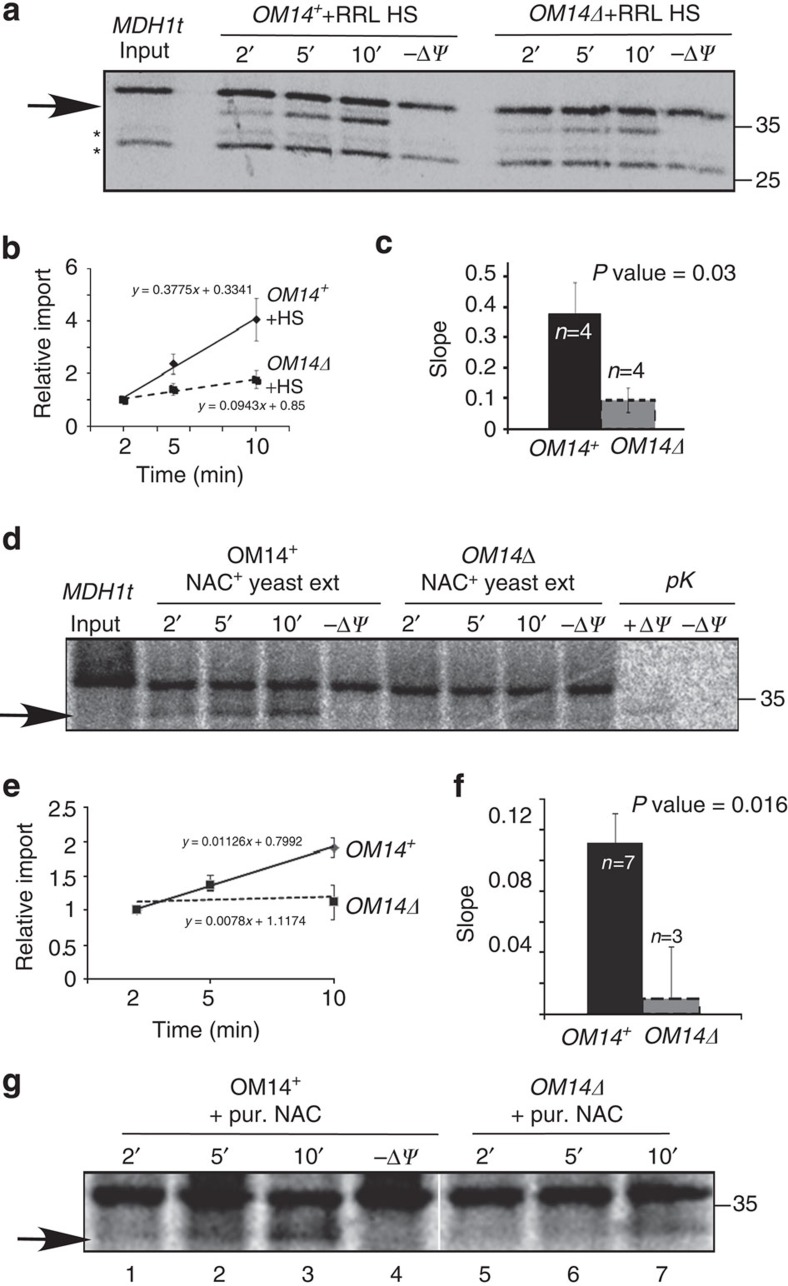
OM14 necessitates NAC to exert its role. (**a**–**c**) NAC from rabbit reticulocyte lysates (RRL): MDH1t RNCs depleted of NAC were mixed with mitochondria purified from either *OM14*^*+*^ or *OM14Δ* cells. The import reactions were supplemented with NAC-containing high-salt supernatant (HS) from RRL, and at the indicated time points a sample was collected. The arrow indicates the imported protein. Import assays were repeated four times, each time with a new mitochondria prep and a new RNC prep. Graphs (**b**), histogram (**c**) and statistics were calculated as described for Fig. 4. (**d**–**f**) NAC from yeast: Import assays entailing NAC-depleted RNCs and OM14^+^ or *OM14Δ* mitochondria were supplemented with ribosome-associated factors, prepared from NAC^+^ yeast strain. Graphs (**e**), histogram (**f**) and statistics were calculated as described for Fig. 4. (**g**) NAC purified from bacteria: Import assays entailing OM14^+^ or *OM14*Δ mitochondria and MDH1t RNCs depleted of NAC, were supplemented with NAC complex that was expressed and purified to a high degree from bacteria (pur. NAC). Samples were collected at the indicated time points, resolved on PAGE and exposed to phosphorimager for 3 days. Panel is from a single image (provided as Supplementary Fig. 4e), which was cropped between lanes 4 and 5 to remove irrelevant lanes.

We then added to the import reaction NAC from three different sources: First, an aliquot of the rabbit reticulocyte lysate high salt wash, which contains NAC, was added. As can be seen in Fig. 5a–c, import was much more significant if mitochondria contained OM14. Second, we purified ribosome-associated proteins (see Methods) either from WT or *NACΔ* yeast cells. The NAC-containing sample significantly improved import over the *NACΔ* extract (Supplementary Fig. 4a, lanes 1–6), consistent with its known role in improving import into mitochondria^24^. This effect, however, was significantly reduced in mitochondria that lack OM14 (Fig. 5d–f and Supplementary Fig. 4a). Third, the NAC complex was purified to a high level from bacteria (Supplementary Fig. 4b), and added to the import reaction (Fig. 5g and Supplementary Fig. 4c,d). The bacterially purified complex improved import efficiency to OM14^+^ mitochondria (Supplementary Fig. 4d lanes 2–7). However, the contribution of NAC to import was much lower when OM14 was absent (Fig. 5g and Supplementary Fig. 4c,d). We note that the latter data are presented only in a qualitative manner as low signals hindered reliable quantification of the impact of the bacterially purified NAC. Nevertheless, in at least three experimental repeats, the same pattern was observed (Supplementary Fig. 4c,d). Thus, the overall data from the three sources of NAC strongly suggest that OM14 exerts its function on mitochondrial import through the NAC complex.

## Discussion

**Figure 6:**
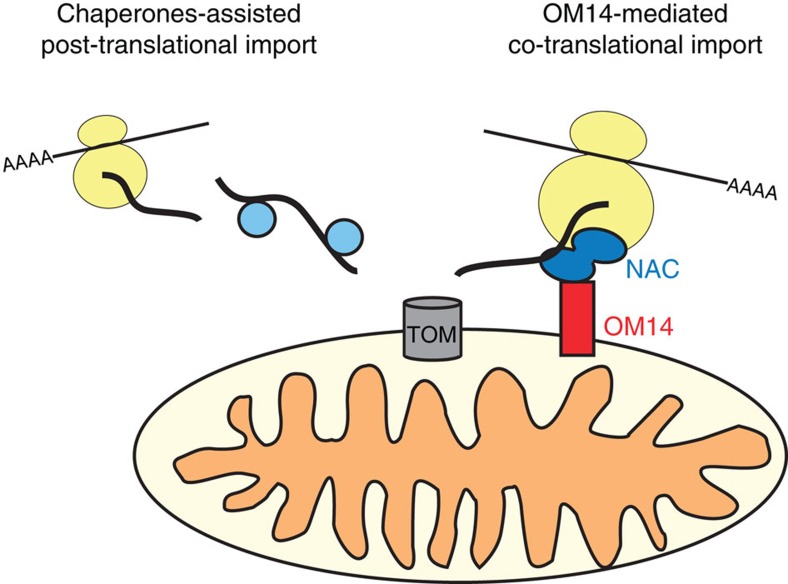
Two modes for protein import into mitochondria. Depicted to the left is the well-established post-translational import mode, in which chaperones (circles) assist fully translated proteins (black line) in their transport through the TOM complex into the mitochondria^49^. Our results corroborate an additional mode, described on the right. In this model, OM14 interacts with the heterodimeric NAC, while it is associated with translating ribosomes. This interaction brings the emerging protein to proximity with the TOM complex thereby enhancing import. These modes are not mutually exclusive and likely to be redundant under many experimental conditions.

Our data provide two critical features in establishing that co-translational import into mitochondria occurs (Fig. 6): first, we identify a novel receptor for NAC (and hence ribosomes) on the mitochondrial outer membrane. Second, we assign a functional significance to this receptor, namely supporting co-translational import into mitochondria.

A receptor for NAC on the mitochondrial outer membrane was proposed 15 years ago^24^, yet was never identified. Our data clearly show that OM14 is critical for NAC association with mitochondria and may serve as its primary receptor. The molecular details of OM14–NAC interaction are yet to be resolved. Interaction may be direct, presumably through the cytoplasmic domains of OM14 (amino acids 1–38 and 90–104; ref. 30) and one of the NAC subunits. Alternatively, OM14 may necessitate an additional protein to stabilize its interaction with NAC, in a mode that resembles the SRP receptor, which is a heterodimer of a small, membrane-embedded protein (SRβ) and a large, mostly cytosolic protein (SRα), which interacts with SRP^38^. We find the latter more likely, and a screen for OM14-interacting proteins is underway.

The clear impact of OM14 deletion on protein import, and the contribution of NAC to this process, strongly suggests that the association between NAC and OM14 is important for co-translational import of proteins into mitochondria (Fig. 6). We suggest that translating ribosomes associate with mitochondria through interaction between NAC and OM14. As NAC interacts with the nascent chains of many proteins, the association with OM14 may be important for initiating specificity for those that are destined to mitochondria. Once association is established, the growing peptide interacts with the import machinery (the TOM complex) and is imported into mitochondria. At least one TOM component (Tom20) was previously shown to be involved in mRNA association with mitochondria, in a translation-dependent manner^10^. Furthermore, the increased levels of Tom20 in *NACΔ* cells (Fig. 3) may indicate a compensation mechanism, which maintains sufficient import efficiency. Import rates in a strain deleted of both Tom20 and OM14 are yet to be determined.

It is difficult to appreciate the relative contributions of post-translational and co-translational protein import to mitochondria. Under standard experimental conditions, when steady-state protein levels are measured, these two processes seem redundant (for example, knockout of key factors does not affect cellular growth). Considering the importance of mitochondrial function to cellular physiology, it is not surprising that cells induce various mechanisms to maintain sufficient amounts of protein in mitochondria. Nevertheless, we speculate that co-translational import is the preferred mode under most natural conditions as it enables efficient and rapid import. Furthermore, it minimizes the chances of protein ectopic expression or the need for an elaborate net of chaperones.

The complete repertoire of proteins that are imported co-translationally is yet unknown. Genome-wide studies of mRNAs association with mitochondria identified many candidates^9^,^10^,^13^,^14^, yet direct impact on mitochondrial activity was rarely demonstrated^39^,^40^. This strongly suggests that both co- and post-translational import mechanisms apply to most proteins, and post-translational import maintains mitochondrial activity when co-translational import is affected. The fact that the same protein can be imported by either way complicates determination of the contribution to the import of each mode. It may, therefore, be necessary to intervene with the post-translational pathway to expose the *in vivo* importance of co-translational import. Nevertheless, we cannot exclude the possibility that co-translational import is applicable only to a subset of proteins. Indeed some specificity in NAC activity was inferred from a genome-wide association study^22^. Thus, NAC may recognize and interact with a subset of emerging peptides. Once NAC interacts with a proper nascent chain, it will be in a better position for interaction with OM14, thereby stabilizing it with the outer membrane.

In this work, we identified a long-sought-after receptor for the ribosome-associated complex NAC. Importantly, we found a physiological role for this receptor, namely import of proteins into mitochondria. The data presented herein are the first to show that localized translation (mediated through NAC–OM14 interaction) supports protein import into mitochondria. The amount of proteins that are targeted by this mode and its implication *in vivo* are yet to be determined. Nevertheless, our data directly link protein synthesis and mitochondria import, thereby providing a strong support to the idea of co-translational import to mitochondria.

## Methods

### Yeast growth and strains

For mitochondrial fractionation and ribosomes isolation, cells were grown in YP-glycerol medium (1% yeast extract, 1% peptone and 2% glycerol). Strains are listed in Table 1.

### Antibodies

The following antibodies were used: polyclonal anti-EGD2 (Rb1) (1:20,000 dilution), (gift from Professor M. Collart)^41^, polyclonal anti-EGD2 (1:20,000 dilution) (gift from Professor B. Beatrix)^42^, monoclonal anti-HA (1:500 dilution) (gift from Professor A. Aronheim)^43^, polyclonal anti-Tom20 (1:10,000 dilution) (gift from Professor D. Rapaport)^44^ and polyclonal anti Rpl3 (1:5,000 dilution) (gift from Professor J. Warner)^45^.

### DHFR-based protein fragment complementation assay

The protein–protein interactions screen was done using the yeast DHFR PCA library according to the published protocol^28^ in 1,536 format. In brief, MATa strains with the ORFs of either Egd1 or Egd2 fused to F[1,2] were mated to the entire MATalpha collection of ORFs tagged with F[3]. The complementary mating was also performed, in which MATalpha strains with the ORFs Egd1 or Egd2 fused to F[3] were mated to the entire MATa collection of ORFs tagged with F[1,2]. The resulting diploids were subsequently selected for growth in the presence of methotrexate for positive DHFR PCA reconstitution for 5 days in 30 °C. The analysis of each plate was done by taking image of the entire plate, which was saved in JPG format at a resolution of 300 d.p.i. Using the freely available Balony software^29^, the area for each colony was extracted and a threshold of a positive interaction was set to be >150.

### Cell fractionation for import analyses

Crude or highly purified mitochondria were isolated as described^36^, with several modifications: Yeast strains were grown in YP-glycerol medium to OD_600_ 1.5–2.0, pelleted, washed and treated with Zymolyase for 30 min at 30 °C with gentle shaking. Spheroplasts were resuspended with 3 ml of ice-cold homogenization buffer (0.6 M Sorbitol, 10 mM Tris HCl pH 7.4, 1 mM EDTA, 1 mM phenylmethyl sulphonyl fluoride (PMSF), 0.2% bovine serum albumin (BSA), and homogenized by 15 strokes using Dounce homogenizer equipped with tight-fitting pestle. Unbroken cells and nuclei were removed by centrifugation for 6 min at 4 °C at 1,500 *g*. The supernatant was then centrifuged for 10 min at 4 °C at 10,000 *g* and the crude mitochondrial pellet was frozen in liquid nitrogen. To obtain highly purified mitochondria, the pellet was washed in SEM buffer (250 mM sucrose, 1 mM EDTA, 10 mM MOPS pH 7.2), resuspended with SEM buffer and layered over four-step sucrose gradient: 1.5 ml 60%, 4 ml 32%, 1.5 ml 23% and 1.5 ml 15% sucrose in EM buffer (10 mM MOPS pH 7.2, 1 mM EDTA). The gradient was centrifuged at 134,000 *g* at 4 °C for 1 h and purified mitochondria were recovered from the 60%/32% interface. Mitochondria were centrifuged again at 10,000 *g* at 4 °C for 10 min, resuspended in SEM buffer and snap frozen in liquid nitrogen and stored at −80 °C. Mitochondria were thawed at 25 °C bath immediately before use and kept on ice.

### Ribosomes purification

Ribosome purification is based on the method described in ref. 46. Specifically, yeast strains were grown in 250 ml YP-glycerol medium to OD_600_ 1.0–1.5. Cells were centrifuged at room temperature for 10 min at 3,000 *g* and the pellet was washed in double distilled water. Cells were resuspended in 1.3 volumes of lysis buffer (Ribo buffer (0.1 M KOAc, 20 mM HepesKOH pH 7.4, 2.5 mM Mg(OAc)_2_), 1 mg ml^−1^ heparin, 2 mM dithiothreitol (DTT), 1 mg ml^−1^ leupeptin, 0.01 mg ml^−1^ Pepstatin A, 1 mM PMSF, 1% Triton X-100) and glass beads (0.4–0.6 mm) were added. Cells were lysed by two pulses of 1.5 min each in Bead Beater and the lysate was clarified by centrifugation in a Sorval SS-34 rotor at 12,000 r.p.m. for 30 min at 4 °C. The supernatant was loaded on 2.5 ml of sucrose cushion (Ribo buffer supplemented with 500 mM KCl, 1 M sucrose and 2 mM DTT) and centrifuged using Beckman Type 70Ti ultracentrifuge rotor at 60,000 r.p.m. for 106 min at 4 °C. The ribosomal pellet was resuspended in 300 μl storage buffer (Ribo buffer supplemented with 250 mM sucrose and 2 mM DTT) and immediately frozen in liquid nitrogen.

Factors that are associated with yeast ribosomes (for Fig. 5d–f) were isolated from ribosomes by loading isolated ribosomes on a high-salt sucrose cushion (25% sucrose, 20 mM HepesKOH pH 7.4, 2 mM DTT, 2 mM Mg(Ac)_2_, 0.8 M KAc, 2.5 μg ml^−1^ leupeptin, 1.8 μg ml^−1^ Pepstatin A, 5 mM PMSF) and centrifugation at 60,000 r.p.m. for 106 min at 4 °C (Beckman Type 70 Ti). The supernatant was frozen in liquid nitrogen and kept at −80 °C.

### Co-immunoprecipitation

Cells were grown in 200 ml YP-glycerol medium to OD_600_ 0.5–1.0, centrifuged at 4 °C for 4 min at 3,000*g* and the pellet was washed twice in buffer A (20 mM Tris HCl pH 8, 140 mM KCl, 1.8 mM MgCl_2_, 0.1% NP-40). Cells were resuspended in 5 ml buffer B (buffer A supplemented with 0.5 mM DTT, 1 mM PMSF, 10 μg ml^−1^ leupeptin, 7 μg ml^−1^ Pepstatin A, 20 units per ml DNase, 1 μg ml^−1^ aprotinin and 10 μg ml^−1^ trypsin inhibitor) and glass beads (0.4–0.6 mm) were added. Cells were lysed by two pulses of 1.5 min each in Bead Beater and the lysate was clarified. One percent of the sample was set aside as ‘Total’ and the rest of the sample was loaded twice on anti-HA-coupled sepharose column and 1% was set aside as ‘FT’ (Flow Through). The column was first washed with buffer B and then twice with washing buffer (100 mM NaCl, 50 mM Tris HCl pH 8, 5% glycerol). Elution sample was collected by adding 0.1 M of acetic acid pH 3. The acetic acid was dissipated and pellets were resuspended in 100 mM Tris HCl pH 8 in 1 × SDS loading dye for western blot analysis.

### Assessing association of ribosomes with mitochondria

Yeast ribosomes were purified as described in the above section (ribosome purification) including a final step of centrifugation in a low-salt sucrose cushion (25% sucrose, 20 mM HepesKOH pH 7.4, 2 mM DTT, 2 mM Mg(Ac)_2_, 0.1 M KAc, 2.5 μg ml^−1^ leupeptin, 1.8 μg ml^−1^ Pepstatin A, 5 mM PMSF). The ribosomal pellet was resuspended in Ribo storage buffer (0.1 M KOAc, 20 mM HepesKOH pH 7.4, 2.5 mM Mg(OAc)_2,_ 250 mM sucrose, 2 mM DTT) and cleared of any membrane compartments or large ribosomal complexes by five rounds of centrifugation at 10,000 *g* for 5 min. The supernatant from the last centrifugation step was immediately frozen in liquid nitrogen. To test the association of ribosomes with highly purified mitochondria, 25 μg of highly purified mitochondria (prepared as described above) were incubated with 10 μg of ribosomes. Incubation was performed for 5 min at room temperature in mitochondria association buffer (MA buffer: 0.6 M sorbitol, 50 mM HepesKOH pH 7.4, 50 mM KCl, 10 mM MgCl_2_, 2 mM KH_2_PO_4_, 1 mg ml^−1^ BSA, 0.75 mg ml^−1^ methionine and fresh 250 mM creatine phosphate and 5 mg ml^−1^ creatine kinase, 2 mM ATP, 8 mM NADH in a total volume of 25 μl). Samples were then centrifuged for 5 min at 10,000 *g*. Supernatant and pellet were subjected to western analysis.

For assessing the association of RNCs containing mitochondrial protein precursor (Fig. 2d), RNCs were generated as described in the section below (Generating ribosome–nascent chains), and 40 μl of RNCs were loaded on 200 μl low-salt sucrose cushion (25% sucrose, 20 mM HepesKOH pH 7.4, 2 mM DTT, 2 mM Mg(Ac)_2_, 0.1 M KAc, 2.5 μg ml^−1^ leupeptin, 1.8 μg ml^−1^ Pepstatin A, 5 mM PMSF), centrifuged using Beckman MicroUltra Centrifuge Rotor TLA-120.2 MicroFixed at 95,000 r.p.m. for 30 min at 4 °C and the RNCs pellet was suspended with 60 μl of MA buffer without BSA. Immediately after isolation, 10 μl of RNCs were incubated with 25 μg of mitochondria for 5 min at room temperature in MA buffer. Samples were then centrifuged for 5 min at 10,000 *g* and the supernatant and pellet were subjected to western analysis.

### Generating ribosome–nascent chains

Plasmid or PCR product containing a truncated MDH1 gene^24^ was introduced into T_N_T SP6 Quick Coupled Transcription/Translation System (Promega L2080). Usually, 1 μg of DNA was mixed with 40 μl of master mix and 2 μl of EasyTag Methionine L-[^35^S]- NEG 709A500UC. The reaction was incubated for 1.5 h at 30 °C. The lysate was loaded on sucrose cushion (25% sucrose, 20 mM HepesKOH pH 7.4, 2 mM DTT, 2 mM Mg(Ac)_2_, 0.1 M KAc, 2.5 μg ml^−1^ leupeptin, 1.8 μg ml^−1^ Pepstatin A, 5 mM PMSF) and centrifuged using Beckman MicroUltra Centrifuge Rotor TLA-120.2 MicroFixed at 95,000 r.p.m. for 30 min at 4 °C. For high-salt wash (Supplementary Fig. 3), the KAc concentration wash increased to 0.8 M. The RNCs pellet was washed and resuspended with 60 μl of import buffer (0.6 M sorbitol, 50 mM HepesKOH pH 7.4, 50 mM KCl, 10 mM MgCl_2_, 2 mM KH_2_PO_4_, 1 mg ml^−1^ BSA, 0.75 mg ml^−1^ methionine and fresh 250 mM creatine phosphate and 5 mg ml^−1^ creatine kinase).

### Co-translational import assay

Import assay was performed essentially as described in ref. 47. Briefly, RNCs were mixed with 25 μg of mitochondria in import buffer supplemented with 2 mM ATP and 8 mM NADH in a total volume of 300 μl. At each time point (2, 5 or 10 min), 100 μl aliquots were transferred to a new ice-cold tube, 1 μg ml^−1^ of valinomycin was added and the sample was kept on ice. Half of each time point sample was treated with 2.5 μg proteinase K for 15 min on ice (proteinase K was inactivated by the addition of PMSF to a final concentration of 2 mM). Samples were centrifuged at 10,000 *g* for 3 min at 4 °C, mitochondria pellets were washed with SEM buffer (250 mM sucrose, 1 mM EDTA, 10 mM MOPS pH 7.2) and resuspended with import buffer without BSA and analysed by polyacrylamide gel electrophoresis (PAGE). Radioactive signals were detected by PhosphorImager and quantified with ImageQuant.

### Purification of yeast NAC complex from bacteria

Purification of yeast NAC complex (EGD2–EGD1) from bacteria was performed essentially as was previously described in ref. 48. Briefly, the dual expression plasmid pOP-H6-EGD2-EGD1 (pA896) was introduced into *Escherichia coli* strain MH1 together with a laq-Iq repressor (pA917) (plasmid and bacteria are a gift from Prof. Elke Deuerling). Cells were grown at 30 °C and NAC expression was induced with IPTG for 3 h. Cell lysate was subjected to initial purification on Ni^+2^ columns (cat. 1018-25, Adar Biotech), utilizing the His_6_ tag present at the amino (N) terminus of EGD2. Eluted material was further purified by gel filtration size-exclusion column (Superdex 200, GE Healthcare) in buffer that contains 20 mM Tris pH 7, 50 mM NaCl and 5 mM MgCl_2_. Both subunits were eluted at a fraction that corresponds to their combined mass (that is, as a formed complex).

### Statistical analyses

Statistical analyses were performed with SPSS. For each strain in each experiment, normality of the results was verified using either Shapiro–Wilk or Kolmogrov–Smirnov tests. Variances were calculated and accordingly *P* values were determined by independent-samples one-sided *t*-test.

## Author contributions

C.L. and Y.A. conceived the project; C.L. performed the Co-IP, ribosomal and mitochondrial association assays and the import assays; Y.C. and M.S. performed and analysed the DHFR PCA screen and the *in vivo* imaging; A.A.-L. performed some of the biochemical purifications; C.L., M.S. and Y.A. wrote the paper. All the authors discussed and analysed the data.

## Additional information

**How to cite this article:** Lesnik, C. *et al.* OM14 is a mitochondrial receptor for cytosolic ribosomes that supports co-translational import into mitochondria. *Nat. Commun.* 5:5711 doi: 10.1038/ncomms6711 (2014).

## Supplementary Material

Supplementary Information Supplementary Figures 1-4

## Figures and Tables

**Table 1:** Yeast strains.

**Description**	**Lab name**	**Genotype**	**Source**
BY4741 (OM14^+^)	yA1	*Mat a, his3*Δ*1, leu2*Δ*0, met15*Δ*0, ura3*Δ*0*	Euroscarf
Egd1 bait in BY4741	yA1224	*MATa Egd1-DHFR F[1,2]::NatR his3*Δ*1 leu2*Δ*0 met15*Δ*0 ura3*Δ*0*	Ref. 28
Egd1 bait in BY4742	yA1225	*MATalpha Egd1-DHFR F[3]::HygroR his3*Δ*1 leu2*Δ*0 lys2*Δ*0 ura3*Δ*0*	Ref. 28
Egd2 bait in BY4741	yA1226	*MATa Egd2-DHFR F[1,2]::NatR his3*Δ*1 leu2*Δ*0 met15*Δ*0 ura3*Δ*0*	Ref. 28
Egd2 bait in BY4742	yA1227	*MATalpha Egd2-DHFR F[3]::HygroR his3*Δ*1 leu2*Δ*0 lys2*Δ*0 ura3*Δ*0*	Ref. 28
OM14Δ	yA1067	*Mat a, his3*Δ*1, leu2*Δ*0, met15*Δ*0, ura3*Δ*0, om14::KanR*	Euroscarf
OM14-HA	yA1144	*Mat a, his3*Δ*1, leu2*Δ*0, met15*Δ*0, ura3*Δ*0, OM14 3XHA- spHIS3*	Homologous recombination with pFA6a-3HA-HIS3MX6 (ref. 50)
NAC^+^	yA318	*Mat a leu2, trp1, his3, ura3, ade2*	Ref. 26
*NAC*Δ	yA321	*Mat a leu2,trp1,his3,ura3,ade2 egd2::ADE2 , egd1::URA3*	Ref. 26
NAC^+^, OM14-HA	yA1156	*Mat a leu2, trp1, his4, ura3, OM14 3XHA- spHIS3*	YA318+ Homologous recombination with pFA6a-3HA-HIS3MX6 (ref. 50)
*NAC*Δ+ OM14-HA	yA1155	*Mat a leu2, trp1, his3, ura3, ade2 egd2::ADE2, egd1::ura3+ OM14 3XHA- spHIS3*	yA321+Homologous recombination with pFA6a-3HA-HIS3MX6 (ref. 50)
*NAC*Δ*, OM14*Δ	yA1167	*Mat a leu2,trp1,om14::spHIS3,egd2::ADE2 , egd1::URA3*	yA321+Homologous recombination with pFA6a-3HA-HIS3MX6 (ref. 50)
