# Supplementary material for: OM14 is a mitochondrial receptor for cytosolic ribosomes that supports co-translational import into mitochondria
Source: Nat Commun. 2014 Dec 9;5:5711. doi: 10.1038/ncomms6711 (PMC4268710; doi:10.1038/ncomms6711)
Supplement: Supplementary Information — Supplementary Figures 1-4 [file ncomms6711-s1.pdf]

Supplementary Figures

a)

b)

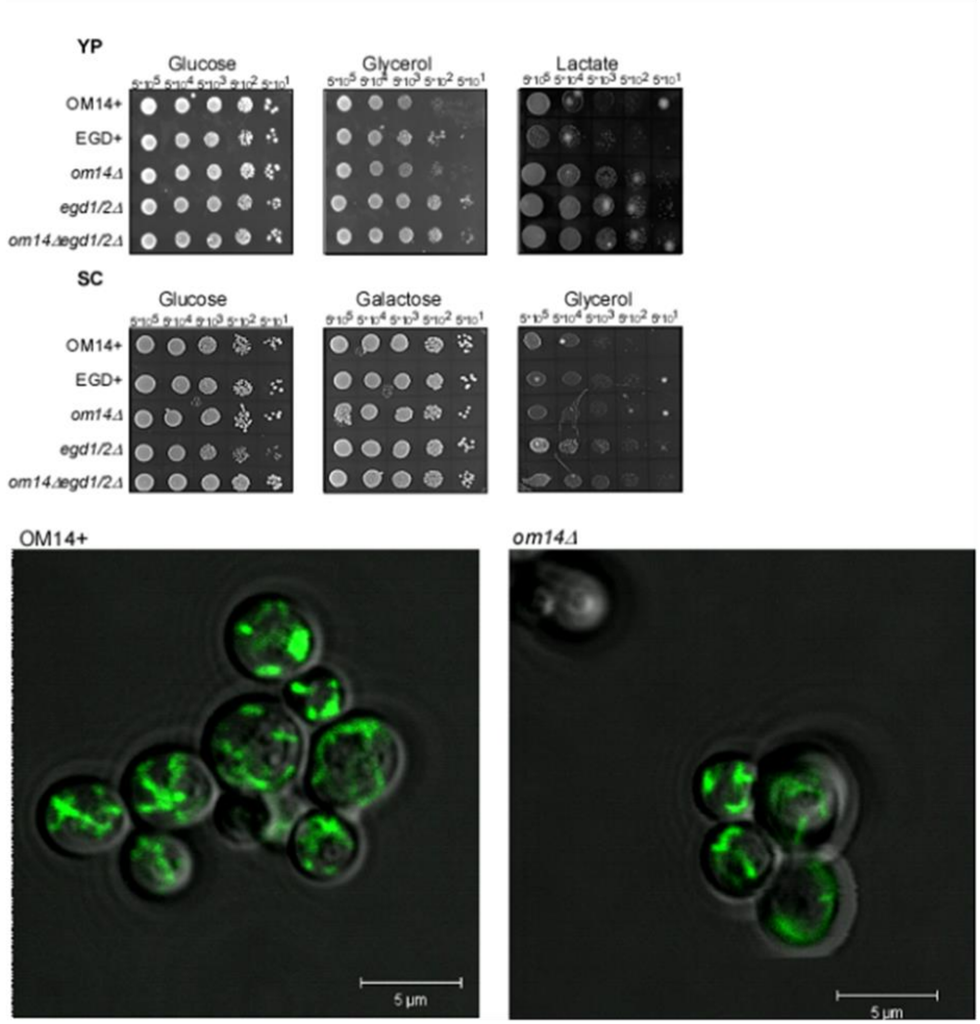

Supplementary Figure 1: OM14 impact on cell growth and protein localization. a) Yeast cells of the indicated lines were spotted in a serial dilution on agar plates with YP supplemented with either 2% glucose, glycerol or lactate (upper panels) or with synthetic complete media supplemented with 2% glucose, galactose or glycerol. Plates were incubated for 4 days. Note that the parental strain for OM14 ($OM14^{+}$) is BY4741 and for EGD is MH272-3fa. b) Plasmid expressing MTS-GFP was introduced either to OM14+ cells or *OM14Δ* cells, and protein localization was monitored. Representative cells are shown, indicating no difference between the lines.

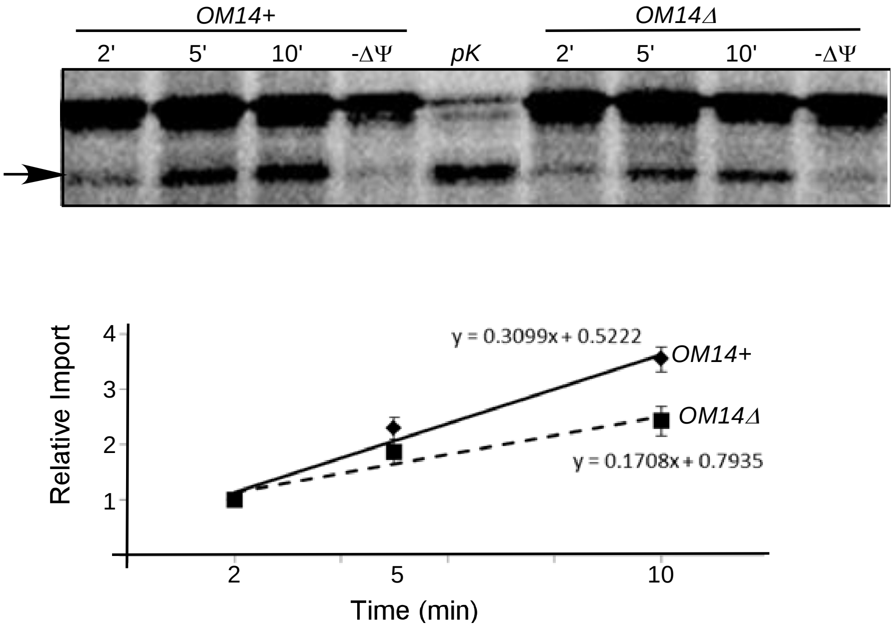

Supplementary Figure 2: Post translational import of Su9-DHFR. The chimeric Su9 MTS-DHFR was synthesized in rabbit reticulocyte lysate in the presence of ^{35}S-Met. Ribosomes and any remaining ribosome-associated protein were removed by centrifugation, and the fully translated protein was mixed with purified mitochondria from OM14+ or *OM14Δ* cells. Upper panel presents the autoradiogram from one import assay, with arrow indicating the imported protein. pK indicates reactions that were subjected to proteinaseK treatment after 10 min of import, and –ΔΨ are 10 min reactions that were performed in the absence of membrane potential. The graphs were derived from at least three independent experiments and error bars are the s.e.m. of each time point. The linear best fit slope is presented. *p* value for the difference between the slopes of the best-fit linear graphs is 0.028.

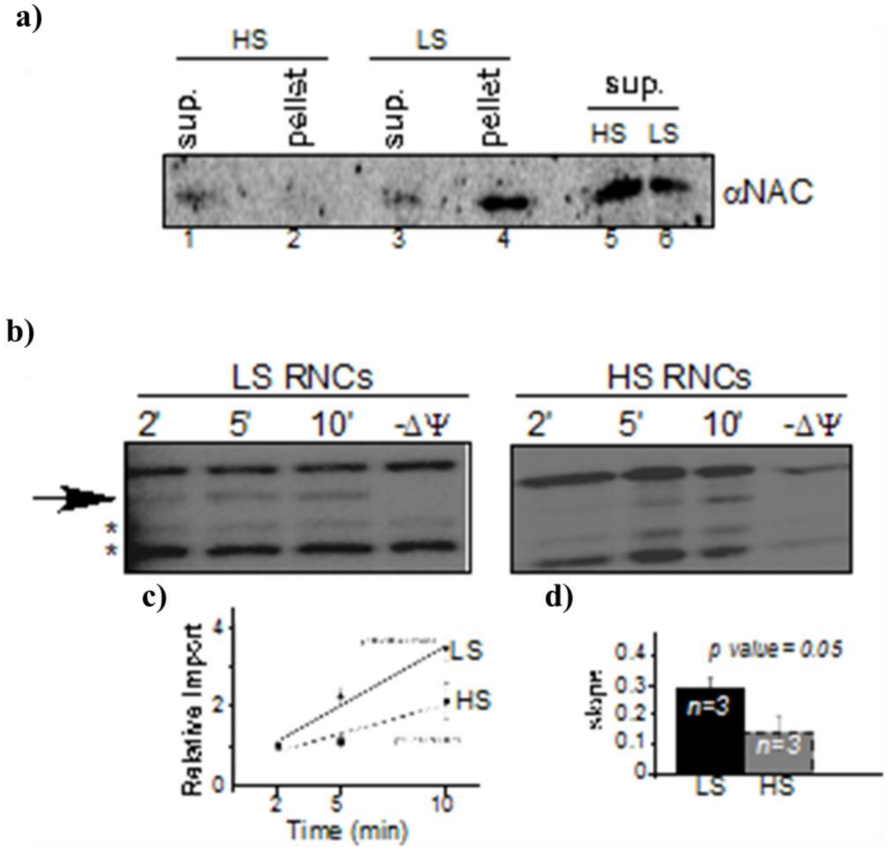

Supplementary Figure 3: High salt striping of NAC reduces import efficiency. a) Western analysis of samples from the NAC depletion procedure. RNCs were centrifuged through either high salt (HS) or low salt (LS) cushion, and samples from the pellet or supernatant (sup.) were analyzed by western analysis (lanes 1-4). Lanes 5 and 6 were loaded with twice the amount of sup. than in lanes 1 and 3, to better visualize the extent of NAC removal.

b) RNCs isolated either through low salt cushion (LS) or through high salt cushion (HS), were mixed with mitochondria from $OM14^+$ cells and import was allowed for the indicated times. Samples were resolved on PAGE and arrow indicates the import product. Asterisks indicate non-specific RNA products.

c-d) The experiments were repeated three times, with three time-points measurements in each. Every repeat entailed a new mitochondria prep and a new RNC prep. The signal corresponding to the imported protein was quantified and the average value and s.e.m. for each time point is presented. Graphs are the best-fit linear slope and Histogram (D) present the difference between the slopes of the LS and HS import assays.

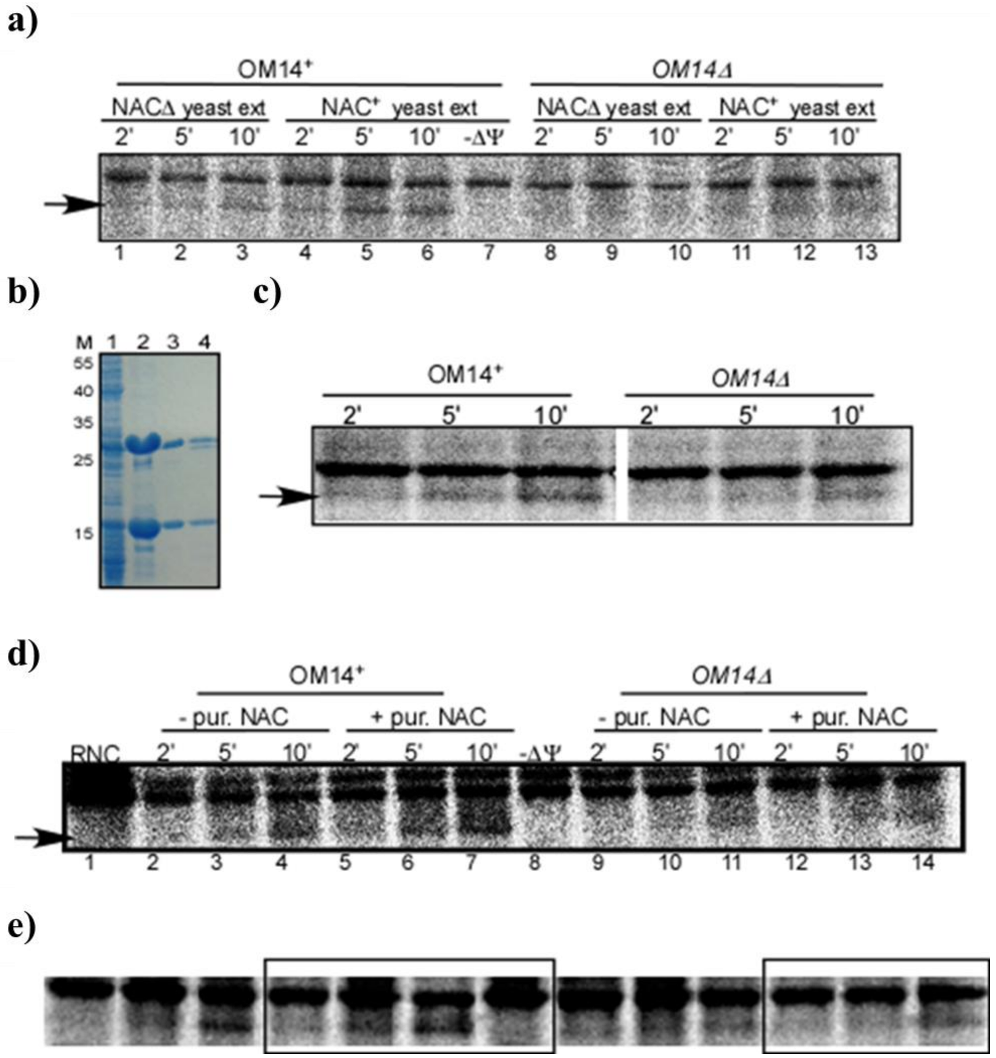

Supplementary Figure 4: Impact of re-addition of NAC on import. a) Import assays entailing $OM14^+$ or *OM14Δ* mitochondria and MDH1t RNCs depleted of NAC were supplemented with ribosome-associated factors, prepared from yeast either deleted of NAC or their parental strain (designated *NACΔ* yeast. ext. and NAC+ yeast ext., respectively.

b) *E. coli* MH1 cells expressing NAC were lysate and NAC was purified through binding to a Nickel column followed by gel filtration. Aliquots were taken from the cell lysate (lane 1), from the elution of nickel column (lane 2), before loading on the gel filtration column (lane 4) and after the elution from the gel filtration column (lane 4), loaded on PAGE and Coomassie blue stained.

c and d) Biological repeats for import assays supplemented with the bacterially purified NAC. Reactions were performed as described for Figure 5 in the main text. Panels are from two different experiments, showing an improved import in the OM14+ mitochondria. $OM14^+$ and *OM14Δ* data are from the same image, that was cropped to remove irrelevant lanes.

e) The original image from which Fig. 5g was made. Boxes indicate the cropped areas.
